# Novel Genetic Variants Associated with Primary Myocardial Fibrosis in Sudden Cardiac Death Victims

**DOI:** 10.1007/s12265-024-10527-5

**Published:** 2024-06-07

**Authors:** Sini Skarp, Anne Doedens, Lauri Holmström, Valerio Izzi, Samu Saarimäki, Eeva Sliz, Johannes Kettunen, Lasse Pakanen, Risto Kerkelä, Katri Pylkäs, Heikki V Huikuri, Robert J Myerburg, Juhani Junttila

**Affiliations:** 1 Research unit of Biomedicine and Internal Medicine, Medical Research Center Oulu, University of Oulu and Oulu University Hospital, Oulu, Finland; 2 Faculty of Biochemistry and Molecular Medicine, University of Oulu, Oulu, Finland; 3 Systems Medicine, Center for Life-Course Health Research, Faculty of Medicine, University of Oulu, Oulu, Finland; 4 Biocenter Oulu, Faculty of Medicine, University of Oulu, Oulu, Finland; 5 Forensic Medicine Unit, Finnish Institute for Health and Welfare, Oulu, Finland; 6 Cancer and Translational Medicine Research Unit, Faculty of Medicine, University of Oulu, Oulu, Finland; 7 Division of Cardiology, Miller School of Medicine, University of Miami, Miami, FL USA

**Keywords:** Sudden cardiac death, Myocardial fibrosis, Genetics

## Abstract

**Abstract:**

Myocardial fibrosis is a common finding in victims of sudden cardiac death (SCD). Whole exome sequencing was performed in 127 victims of SCD with primary myocardial fibrosis as the only pathological finding. These cases are derived from the Fingesture study which has collected data from autopsy-verified SCD victims in Northern Finland. A computational approach was used to identify protein interactions in cardiomyocytes. Associations of the identified variants with cardiac disease endpoints were investigated in the Finnish national genetic study (FinnGen) dataset. We identified 21 missense and one nonsense variant. Four variants were estimated to affect protein function, significantly associated with SCD/primary myocardial fibrosis (Fingesture) and associated with cardiac diseases in Finnish population (FinnGen). These variants locate in cartilage acidic protein 1 (CRATC1), calpain 1 (CAPN1), unc-45 myosin chaperone A (UNC45A) and unc-45 myosin chaperone B (UNC45B). The variants identified contribute to function of extracellular matrix and cardiomyocytes.

**Graphical Abstract:**

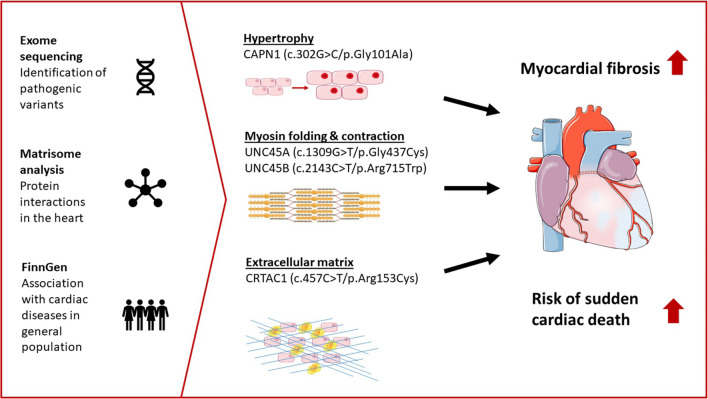

**Supplementary Information:**

The online version contains supplementary material available at 10.1007/s12265-024-10527-5.

## Introduction

Sudden cardiac death (SCD) is the most common mode of death in Western Societies, accounting for ~375,000 deaths in the US annually and approximately 50% of all deaths related to cardiovascular diseases (CVD) [1]. In post-mortem investigations, myocardial fibrosis is a common finding, and a majority of SCD victims have fibrotic accumulations in the myocardium, regardless of whether the underlying cardiac disease is ischemic or non-ischemic [2]. Studies have also demonstrated that myocardial fibrosis detected by cardiac magnetic resonance imaging (MRI) is a powerful predictor of future arrhythmic events and SCD [3, 4].

Coronary artery disease, acquired non-ischemic myocardial diseases and inherited cardiomyopathies such as hypertrophic cardiomyopathy (HCM), dilated cardiomyopathy (DCM), and arrhythmogenic right ventricular cardiomyopathy (ARVC) are common causes of SCD [5]. Despite cardiomyopathies having unique phenotypic manifestations and disease trajectories, myocardial fibrosis is present in every entity, often expressing as the first marker of subclinical disease [6]. In our previous studies on autopsy verified SCD victims in Northern Finland (Fingesture study), we have found that fibrotic accumulations in the myocardium without apparent cause or phenotypic expression of HCM, DCM, or ARVC i.e. primary myocardial fibrosis (PMF), is also frequently observed in young SCD victims [7] and that these cases share genetic variants with HCM, DCM, and ARVC [8]. Previously, variants predisposing to SCD have been identified in genes associated with cardiomyopathy, CAD and inherited arrhythmia syndromes [9]. However, despite the high suspicion of genetic etiology, a majority of the SCDs due to myocardial diseases have not been linked with recognizable disease-causing genetic variants in cardiomyopathy-associated genes [8, 10]. Genetic testing enables identifying at-risk family members of the SCD victim , as well as improving the knowledge of disease pathophysiology, which can lead to discovery of novel targets for therapeutic interventions.

In the present study, we performed exome sequencing in tissue from non-ischemic SCD victims with PMF at autopsy with the goal of seeking novel candidate genes and variants associated with the development of myocardial fibrosis. Identifying genetic variants associated with PMF not only sheds light on the phenotypic expression observed in SCD victims but also holds the potential to enhance the understanding of the broader mechanisms underlying myocardial fibrosis. Additionally,we investigated the biochemical functions and analyzed interactions of detected variants within the matrisome i.e. with the genes encoding ECM and ECM-associated proteins [11], and validated the findings in a large prospective general population cohort (FinnGen).

## Methods

### The Fingesture Study

**Fig. 1:**
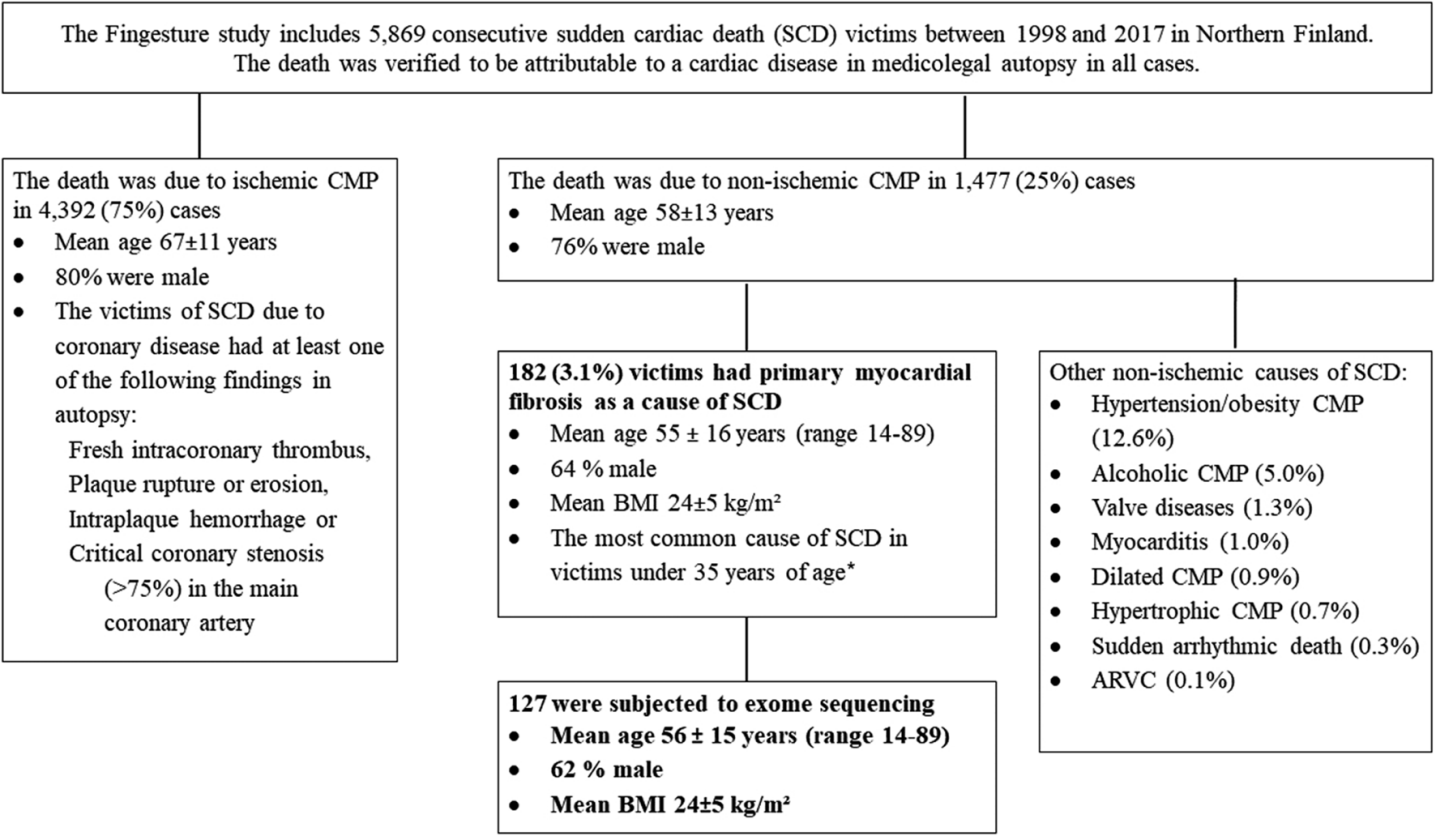
Description of the medicolegal autopsy findings in the Fingesture study. The Fingesture study includes 5869 consecutive sudden cardiac death (SCD) victims. In 25% the cause was non-ischemic and exome sequencing was performed for 127 victims with primary myocardial fibrosis as a cause of non-ischemic SCD. Abbreviations: ARVC = Arrhythmogenic right ventricular cardiomyopathy, CMP = Cardiomyopathy. * Hookana et al Heart Rhyhtm 2012, Vähätalo et al. Cardiology 2022

The Fingesture study (Finnish Genetic Study for Arrhythmic Events) has collected clinical and autopsy data from 5869 successive sudden cardiac death (SCD) victims from 1998 to the present from the geographically-defined area of the Oulu University Hospital District in Northern Finland (Fig 1). The complete study protocol has previously been published [7]. In short, a medicolegal autopsy was performed on all SCD victims in the Department of Forensic Medicine, University of Oulu, Oulu, Finland, or in the Finnish for Health and Welfare, Oulu, Finland, by experienced forensic pathologists, each performing more than 100 autopsies per year, making use of contemporary guidelines for diagnosis of the cause of death [12]. The Finnish law requires a medicolegal autopsy if the death cannot be attributed to a known disease, if the victim was not treated by a physician during his/her last illness, or if the death was in other ways unexpected. The hearts of the SCD victims were meticulously inspected, including measurements of cardiac weight, macroscopic dissection and examination of the myocardium, coronary arteries and valves, and histological examination of 3 - 5 myocardial samples. The determination of myocardial fibrosis was based on macroscopic and histological investigation of myocardial tissue at autopsy.

### Study Population

We studied 127 victims of nonischemic SCD with PMF as the only pathological finding. The 127 samples were selected from a total 182 based on quality of the sample and DNA. The mean age of study population was 56 years (range 14-89, SD=15.1). There are total 71 SCD victims under 35 year-old in the Fingesture cohort (*N*=5869) and 11 of them are included in the exome sequencing. PMF was defined by the replacement of myocytes with patchy, interstitial, or diffuse fibrous tissue in the absence of chronic disease of coronary arteries, healed myocardial infarction, anatomy associated with inherited structural cardiac diseases (DCM, HCM, ARVC), cardiac valve disease, myocarditis, or hypertensive left ventricular hypertrophy (HLVH) with or without secondary scarring. To minimize HLVH, only individuals with a cardiac weight below 420 grams, and with absence of hypertrophied myocytes, were classified as PMF. Other noncardiac diseases or organ changes that are associated with myocardial fibrosis such as myotonic dystrophy, systemic sclerosis, or Fabry disease, were absent.

### Whole Exome Sequencing and Data Analysis

DNA isolated from formalin fixed and paraffin embedded (FFPE) myocardial tissue samples from 127 PMF cases was used for whole exome sequencing (DNA Prep with Enrichment Library Kit and NextSeq550 platform, Illumina). Recent comparison of exome sequencing of FFPE samples and blood samples of sudden death cases showed that exome sequencing is both sensitive and accurate compared to blood samples leading to only small number of false positive or false negative variant calls [13]. Sequence alignment and variant calling was conducted in BaseSpace Genomics computing environment (Illumina) using BWA Genome Aligner and the GATK Variant Caller. Variant Interpreter and wANNOVAR were used for annotation and filtering of variants. In order to filter out likely benign variants the variant effect was estimated by filtering the variant files using the following criteria: The variant had to be rare (MAF < 0.005). Allele frequencies of the Finnish population were obtained from GnomAD and ExAC (gnomad.broadinstitute.org) and of the local population in Northern Ostrobothnia from the FINRISK database (sisuproject.fi). The variant had to have the following functional estimations: missense variants had to be classified as deleterious by SIFT and possibly or probably damaging by PolyPhen-2, CADD score > 20 and indels that were predicted to be disease causing by MutationTaster were included. For Varity and MutScore estimates a score of > 0.7 is considered a deleterious estimation. The cDNA and protein sequences for transcripts were obtained using Ensembl genome browser and NCBI databases. Variants located in typical false positive genes were automatically discarded [14]. Integrative Genomics Viewer (IGV) was used for data visualization to exclude falsely annotated variants and sequencing artifacts.

### Statistical Analyses

The odds ratio (OR) was used in statistical analyses to assess the association between the variants and myocardial fibrosis. To calculate the allelic OR, the allele count of the variant of interest in the PMF population times the allele count of the reference allele in the general population was divided by the allele count of the variant of interest in the general population times the allele count of the reference allele in the PMF population. Allele counts were estimated using the variant frequency of the whole exome sequencing (WES) data set and the frequency of the Northern Ostrobothnia province (Finrisk). If the frequency of the Northern Ostrobothnia province was unavailable, the general frequency in Finland was used derived primarily from Finrisk and secondarily from gnomAD.

### Gene Classification and Matrisome Analysis

The identified genes were classified according to their literature-based function and expression in the heart. To gain an understanding of the function of the variants, a computational approach was used to model the biochemical effect of the variants and protein interactions in cardiomyocytes. To achieve this aim variants were classified as transversions or transitions and the amino acids targeted by the variants were classified based on their chemical (aliphatic, aromatic, neutral, acidic, basic or unique) and physical (electrical dipole and size) characteristics. The amino acid positions targeted by the variants were also scanned for known post-translational modifications (phosphorylation, acetylation, methylation, N- and O-glycosylation, ubiquitilation, sumoilation and hydroxylation) as reported. At the protein level, the variants were mapped into their Pfam domain of origin, if any, and enriched for antibody staining profile (roughly corresponding to their protein abundance in tissue) in cardiomyocytes from heart tissue using data from The Human Protein Atlas (proteinatlas.org) . A compendium of known interactors of any protein targeted by variants was compiled from BioGRID (thebiogrid.org) and the Human Reference Interactome (interactome-atlas.org) subsetting the former to physical multi-validated interactions. Interactions for the proteins targeted by the variants were then catalogued according to various criteria, such as their enhancement in the heart (high or medium intensity-only staining profiles from The Human Protein Atlas for the interactors considered) and/or belonging to the extracellular matrix (ECM)/matrisome [11].

### Associations of Identified Variants in Cardiac Disease Endpoints

Associations of identified variants were investigated in the FinnGen dataset. Our aim was to investigate if the identified variants would also associate to cardiac diseases in general Finnish population. The FinnGen project is a biobank study that aims to collect and analyse genome and health data from 500 000 Finnish biobank participants (www.finngen.fi). The FinnGen study has generated genetic association data of 309,154 Finnish individuals and 3,095 endpoints (data freeze 7). The endpoints in FinnGen have been defined based on national hospital discharge, death, and medication reimbursement registries and diagnoses are based on the International Classification of Diseases (ICD) codes. The methods of FinnGen project is described in detail by Kurki et al^.^ [15] Shortly, association analyses were performed using SAIGE mixed models with age, sex and 10 PCs used as covariates. We examined the associations between the variants identified in WES analysis and cardiac disease endpoints in FinnGen (Supplementary Table 1). The genetic association results were derived from the FinnGen study database of GWAS summary statistics. A detailed description of all endpoints can be found from the FinnGen phenotype database (https://r7.risteys.finngen.fi/).

The patients and control subjects in FinnGen provided an informed consent for biobank research, based on the Finnish Biobank Act. Alternatively, older research cohorts, collected before the start of FinnGen (in August 2017), were collected on the basis of study-specific consents and later transferred to the Finnish biobanks after approval by Fimea, the National Supervisory Authority for Welfare and Health. Recruitment protocols followed the biobank protocols approved by Fimea.

## Results

### Identifying Predisposing Variants

**Fig. 2:**
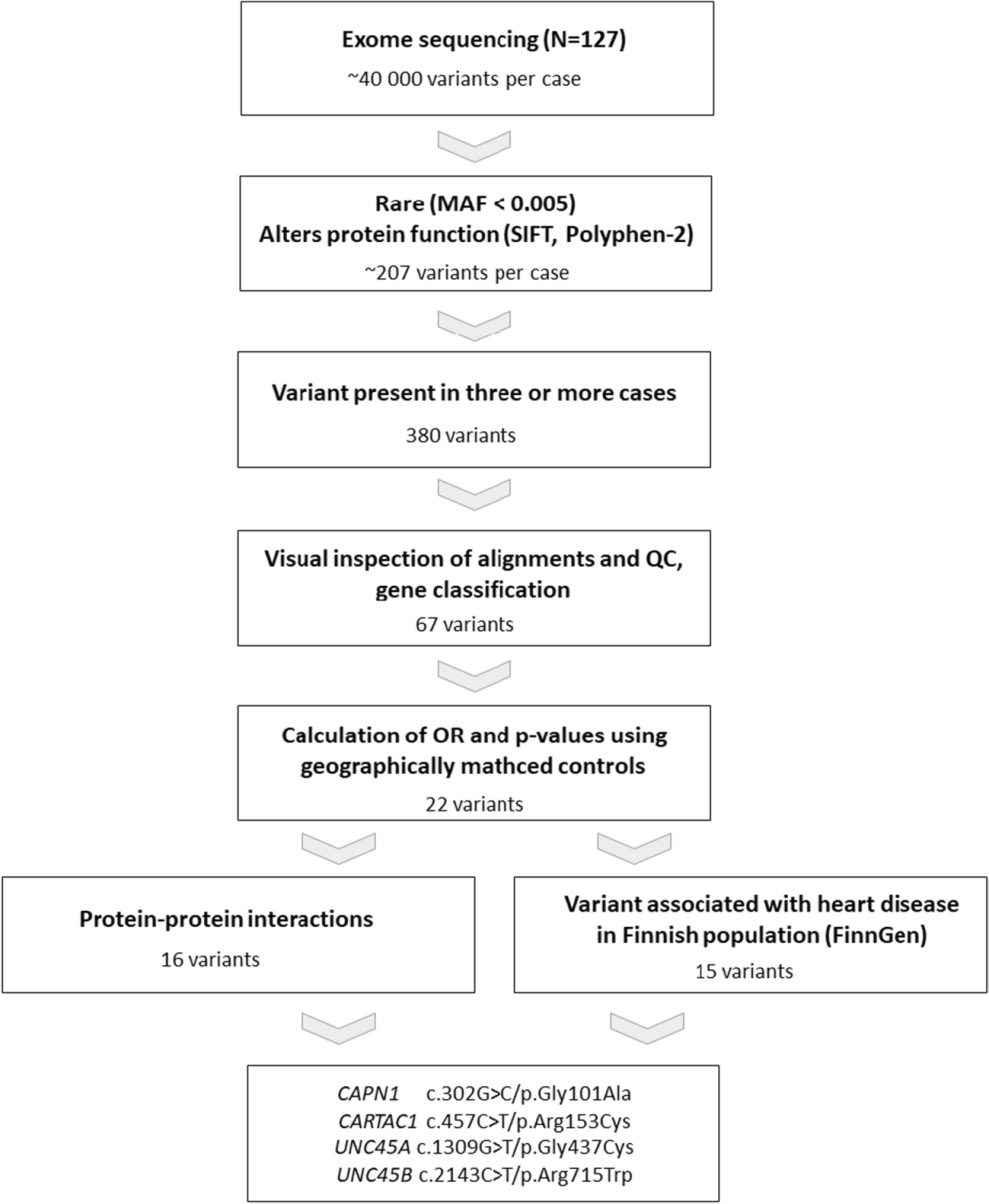
Overview of the variant analysis. The flowchart represents the steps involved in performing the variant analysis

The mean age of the study subjects was 56.3±15.1 years and 37.8% were women. The mean BMI was 24.4±4.8 kg/m^2^ (Table 1). The exome sequencing detected more than 40,000 variants per case with average coverage of 89.95X of the target (45 Mb). Out of these, we identified 22 rare variants (MAF < 0.005), present in at least three cases, that were estimated to be functional harboring protein-altering variants with missense variants further predicted to be deleterious (Table 2, Fig. 2). At least one of the 22 identified variants was present in 54 cases (43% of the 127 studied). The mean age of these variant-carrying cases was 54.2 ± 15.4 years (range 14-85) and six were under 35. The identified variants include 21 missense variants in 21 genes and one non-sense variant in the *HPSE* gene. Several of these variants are in genes participating in ECM formation or have been previously associated with heart function features, such as contractility, arrhythmias, hypertrophy or ischemic CVD (Table 2). All variants were heterozygous. Additionally, we identified three pathogenic variants reported in ClinVar database, each in a single individual (Supplementary Table 2). Two of these variants are associated with hypertrophic cardiomyopathies and one with sitosterolemia.

**Table 1:** Demographics and clinical characteristics of study subjects (*n*=127). Continuous variables are presented as mean±SD

Characteristic	Value
Age, years	56.3±15.1
Sex, *n* (%)	
Women	48/127 (37.8%)
Men	79/127 (62.2%)
BMI, kg/m^2^	24.4±4.8
Time of SCD, *n* (%)	
12am-6am	12/41 (29.3%)
6am-12pm	10/41 (24.4%)
12pm-6pm	10/41 (24.4%)
6pm-12am	9/41 (22.0%)
SCD during physical exercise, *n* (%)	12/70 (17.1%)
Pre-SCD clinical characteristics, *n* (%)	
Diabetes mellitus	12/123 (9.8%)
Heart failure	0 (0%)
Hypertonia	22/123 (17.9%)
Dyslipidemia	10/123 (8.1%)
Dyspnea	1/123 (0.8%)
Angina	3/123 (2.4%)
Degree of myocardial fibrosis	
Substantial	3/127 (2.4%)
Moderate patchy	54/127 (42.4%)
Scattered mild	70/127 (55.1%)
None	0 (0%)

**Table 2:** Variants identified in whole exome sequencing. A variant is marked with underlining if it has Varity_R or MutScore > 0.7. Benjamini Hochberg method was used to adjust for multiple test correction

Gene	Variant	dbSNP	GnomAD FIN	FINRISK	N cases (AF)	Functional Prediction	OR (CI)	*P*	Adj. P
Extracellular matrix									
*HPSE*	c.1294C>T/ p.Arg432Ter	rs141973931	0.0045	0.0026	3 (0.012)	MT=DC; CADD=44.0	4.52 (1.16 - 17.59)	0.029	0.054
*CRTAC1*	c.457C>T/ p.Arg153Cys	rs140424345	0.0041	0.0023	3 (0.012)	SIFT=D, PP2=PD, VR=0.386, MS=0.764, CADD=32.0	5.27 (1.31 - 21.22)	0.019	0.042
*NMRK2*	c.242C>T/ p.Pro81Leu	rs146474422	0.0049	0.0042	3 (0.012)	SIFT=D, PP2=PD, VR=0.286, MS=0.508 CADD= 23.8	2.85 (0.79 - 10.28)	0.110	0.126
*LIMS1*	c.301G>A/ p.Ala101Thr	rs145559017	0.0030	0.0005	3 (0.012)	SIFT=D, PP2=PD, VR=0.258, MS=0.186 CADD=27.1	22.65 (2.35 - 218.58)	0.007	0.019
*FRAS1*	c.10426G>A/ p.Val3476Met	rs201963922	0.0011	0.0012	3 (0.012)	SIFT=D, PP2=PD, VR=NA, MS=0.421, CADD=25.4	10.33 (2.07 - 51.45)	0.004	0.014
*TGM6*	c.419G>A/ p.Cys140Tyr	rs146485197	0.0029	0.0049	3 (0.012)	SIFT=D, PP2=PoD, VR=0.38, MS=0.362, CADD= 25.3	2.43 (0.69 - 8.57)	0.169	0.169
*TNS2*	c.3848G>A/ p.Gly1283Asp	rs200670407	0.0038	0.0027	3 (0.012)	SIFT=D, PP2=PD, VR=0.94, MS=0.638, CADD= 28.6	4.52 (1.16 - 17.57)	0.029	0.054
Cardiomyocytes/Contractility									
*MYBPHL*	c.193C>T/ p.Arg65Trp	rs146641385	0.0042	0.0042	3 (0.012)	SIFT=D, PP2=PoD, VR=0.35, MS=0.446, CADD= 23.9	2.87 (0.8 - 10.36)	0.107	0.126
*UNC45B*	c.2143C>T/ p.Arg715Trp	rs141654082	0.0008	NA	3 (0.012)	SIFT=D, PP2=PD, VR=0.783, MS=0.785, CADD= 25.5	14.98 (4.42, 50.73)*	1.7x10^-5^	0.0004
*UNC45A*	c.1309G>T/ p.Gly437Cys	rs146513919	0.0014	0.0019	4 (0.015)	SIFT=D, PP2=PD, VR=0.896, MS=0.873, CADD= 31.0	8.48 (2.26 - 31.77)	0.002	0.011
Cardiac hypertrophy									
*CAPN1*	c.302G>C/ p.Gly101Ala	rs534135243	0.0019	0.0019	3 (0.012)	SIFT=D, PP2=PD, VR=0.941, MS=0.922, CADD= 31.0	6.33 (1.5 - 26.65)	0.012	0.029
*NRIP1*	c.717A>T/ p.Arg239Ser	rs202001270	0.0015	0.0049	3 (0.012)	SIFT=D, PP2=PD, VR=0.937, MS=0.573, CADD=23.6	2.43 (0.69, 8.57)	0.169	0.169
Cardiac Mitochondria									
*OMA1*	c.1093G>T/ p.Asp365Tyr	rs77980955	0.0029	0.0038	3 (0.012)	SIFT=D, PP2=PD, VR=0.857, MS=0.858, CADD= 28.2	3.16 (0.86 - 11.56)	0.082	0.120
*HK2*	c.1057C>T/ p.Arg353Cys	rs61748096	0.0041	0.0030	3 (0.012)	SIFT=D, PP2=PD, VR=0.336, MS=0.242, CADD= 23.3	3.95 (1.04 - 15)	0.043	0.073
Immunity/Inflammation									
*TLR3*	c.889C>G/ p.Leu297Val	rs35311343	0.0009	0.0008	3 (0.012)	SIFT=D, PP2=PD, VR=0.637, MS=0.156, CADD= 22.9	15.85 (2.64 - 95.3)	0.003	0.011
*CCL22*	c.230C>A/ p.Ala77Asp	rs777890031	0.0013	0.0042	3 (0.012)	SIFT=D, PP2=PD, VR=0.787, MS=0.522, CADD= 23.3	2.87 (0.8 - 10.36)	0.107	0.126
Other									
*SYT9*	c.1057C>G/ p.Leu353Val	rs117876446	0.0039	0.0043	3 (0.012)	SIFT=D, PP2=PD, VR=0.457, MS=0.442, CADD=25.0	2.81 (0.78 - 10.14)	0.114	0.126
*MTUS2*	c.637C>T/ p.Arg213Trp	rs201600406	0.0049	0.0035	5 (0.019)	SIFT=D, PP2=PoD, VR=NA, MS=NA, CADD= 22.5	5.75 (1.91 - 17.3)	0.002	0.011
*PLB1*	c.2698G>A/ p.Val900Met	rs142314104	0.0004	0.0008	3 (0.012	SIFT=D, PP2=PD, VR=0.256, MS=0.05, CADD= 25.4	15.85 (2.64 - 95.3)	0.003	0.011
*WNT8B*	c.359G>T/ p.Gly120Val	rs200171146	0.0016	0.0012	3 (0.012	SIFT=D, PP2=PD, VR=0.963, MS=0.868, CADD= 28.8	10.34 (2.08 - 51.49)	0.004	0.014
*VASN*	c.889C>T/ p.Arg297Cys	rs148092711	0.0038	0.0036	3 (0.012	SIFT=D, PP2=PoD, VR=0.109, MS=0.241, CADD= 26.2	3.3 (0.99 - 11.04)	0.052	0.082
*GPR17*	c.428T>C /p.Met143Thr	rs202004329	0.0045	0.0039	3 (0.012	SIFT=D, PP2=PD, VR=0.916, MS=0.763, CADD= 26.4	3.07 (0.84 - 11.24)	0.089	0.123

*D* Deleterious, *DC* Disease causing, *MS* MutScore, *MT* Mutation taster, *PD* Probably damaging, *PoD* Possibly damaging, *PP2* PolyPhen-2, *VR* Varity_R, *GnomAD FIN* Minor allele frequency in GnomAD Finnish population, *OR* Allelic Odds Ratio, *CI* Confidence interval, *= in calculating OR GnomAD FIN used in stead of Finrisk

Ten of the 22 variants were significantly associated with SCD and PMF (Table 2). The matrisome analysis identified ECM related protein-protein interactions for most of these genes (Supplementary Table 3). Nine missense variants had Varity_R or MutScore probability higher than 0.7 and five of these (located in *CAPN1*, *CRTAC1*, *UNC45A*, *UNC45B*, *WNT8B*) were significantly associated with SCD and PMF (Table 2). Indicating that these five variants are the most likely candidates to alter protein function.

### Heart Enhanced Matrisome Interactions

The matrisome analysis identified heart enhanced protein-protein interactions for 16 of the 22 proteins carrying the identified variants (Supplementary Table 3). Four of the five variants having significant association with SCD and PMF (Table 2) were predicted deleterious by computational algorithms and were located in proteins that had heart enhanced interactions according to the Human Protein Atlas. CRATC1 has heart enhanced interaction with Microtubule Associated Protein RP/EB Family Member 2 (MAPRE2). UNC45A and UNC45B have heat shock proteins HSP90AA1 and HSP90AB1 as heart enhanced interactors. UNC45A was also observed to interact with Tektin 1 (TEKT1) and Family With Sequence Similarity 83 Member H (FAM83H) and UNC45B with Activator Of HSP90 ATPase Activity 1 (AHSA1). CAPN1 had heart enhanced interactions with 32 proteins including Actin Alpha Cardiac Muscle 1 (ACTC1), actin binding protein Alpha-Actinin-2 (ACTN2) and AKT serine/threonine kinase 1 (AKT1). No interactions were observed for WNT8B. Of the other genes that had significant association with myocardial fibrosis, LIMS1, TLR3 and PLB1 had heart enhanced interactions. LIMS1 had intractions with nine proteins including Actin Beta (ACTB) and integrin-linked protein kinase (ILK). TLR3 was observed to interact with 20 proteins. These included for example voltage-gated potassium channels (KCND2, KCND3) and components of the MAP kinase signal transduction pathway (MAP2K6, MAP3K7). PLB1 had heart enhaced interaction with TRNA Splicing Endonuclease Subunit 15 (TSEN15). No interactions were observed for MTUS2 or FRAS1.

The genes carrying these variants do not seem to target preferentially specific matrisome categories (core matrisome vs. matrisome-associated, Chi-square test *P* value: 0.615) or families (basement membranes, collagens, ECM glycoproteins, ECM regulators and secreted factors, Chi-square test *P* value: 0.672). There is, conversely, a significant enrichment for interactions with heart-enhanced proteins in this set than expected by chance among all heart-enriched proteins (hypergeometric test *P* value < 10^-16^), suggesting an important role for the proteins targeted by these variants as organizers of the heart interactome i.e. the network of functional interactions between molecules within the heart.

### Associations of Candidate Variants in FinnGen

Four variants estimated to be funcitonal and significantly associated with PMF in Fingesture were also associated with cardiac disease in FinnGen. Altogether 15 of 22 variants had associations with cardiac disease endpoints in FinnGen. Detailed association results are presented in the additional material (Supplementary Table 4). Associations with cardiomyopathies, heart failure and myocarditis are summarized in Table 3. Four variants, c.302G>C/p.Gly101Ala (*CAPN1*), c.637C>T/p.Arg213Trp (*MTUS2*), c.457C>T/p.Arg153Cys (*CRTAC1)*, c.1309G>T/p.Gly437Cys (*UNC45A*), were associated with cardiomyopathies. The variant in *CAPN1* and 3 other missense variants, c.3848G>A/p.Gly1283Asp (*TNS2*), c.419G>A/p.Cys140Tyr (*TGM6*), c.230C>A/p.Ala77Asp (*CCL22*) and c.2143C>T/p.Arg715Trp (*UNC45B*), were associated with heart failure. The nonsense variant c.1294C>T/p.Arg432Ter in *HPSE* as well as the missense variant in *MTUS2* are associated with myocarditis.

**Table 3:** Summary of variant associations to cardiomyopathies, heart failure and myocarditis in Finnish population. A variant is marked with underlining if it is considered likely to alter protein funciton based on in silico estimations and significant association with SCD and PMF

Phenotype category	Variant	Gene	*P* value
Cardiomyopathies	c.302G>C/p.Gly101Ala; rs534135243	*CAPN1*	0.005
	c.637C>T/p.Arg213Trp; rs201600406	*MTUS2*	0.005
	c.457C>T/p.Arg153Cys; rs140424345	*CRTAC1*	0.003
	c.1309G>T/p.Gly437Cys; rs146513919	*UNC45A*	0.036
Heart failure	c.302G>C/p.Gly101Ala; rs534135243	*CAPN1*	0.005
	c.3848G>A/p.Gly1283Asp; rs200670407	*TNS2*	0.014
	c.419G>A/p.Cys140Tyr; rs146485197	*TGM6*	0.020
	c.230C>A/p.Ala77Asp; rs777890031	*CCL22*	0.047
	c.2143C>T/p.Arg715Trp; rs141654082	*UNC45B*	0.049
Myocarditis	c.637C>T/p.Arg213Trp; rs201600406	*MTUS2*	0.014
	c.1294C>T/p.Arg432Ter; rs141973931	*HPSE*	0.018

## Discussion

Among 127 SCD victims with confirmed myocardial fibrosis, we identified 22 variants in an exome sequencing data set. Most variants were located in genes participating in ECM formation, inflammation, mitochondrial function, cardiomyocyte contractility or hypertrophy. None of these variants have been previously reported in association with human disease. Ten variants were significantly associated with PMF. Sixteen of 22 variants were also associated with cardiac diseases in the general Finnish population (FinnGen). Five missense variants were both likely to affect gene function and significantly associated with PMF. Furthermore, four of these were also associated with cardiac diseases in the general Finnish population. These variants locate in cartilage acidic protein 1 (*CRATC1*), the calpain 1 (*CAPN1*), unc-45 myosin chaperone A (*UNC45A*) and unc-45 myosin chaperone B (*UNC45B*). Overall, we identified novel candidate variants and genes predisposing to PMF in SCD victims.

The accumulated ECM in the myocardium is largely produced and remodeled by myofibroblasts. The missense variant c.457C>T/p.Arg153Cys in *CRATC1* was estimated to affect protein funciton and was significantly associated with PMF in Fingesture. Furthermore, this variant was associated with cardiomyopathy in FinnGen (*p*=0.003). Accumulation of fibrosis is an established hallmark of cardiomyophathies. CRTAC1 is a secreted protein that participates in cell-cell and cell-matrix interactions in ECM. CRATC1 was detected to have heart enhanced interaction with MAPRE2, which is associated with cardiac arrhythmia disorder, Brugada syndrome [16]. A study of human dermal fibroblasts demonstrated that CRATC1 stimulates fibroblast proliferation and collagen expression suggesting that it might induce pro-fibrotic pathways [17].

In addition to fibroblasts, cardiomyocytes also play an important role in the formation of fibrosis. Death or damage of cardiomyoctes triggers secretion of pro-fibrotic inflammatory factors [18]. We identified a missense variant c.302G>C/p.Gly101Ala in the *CAPN1* gene*,* which encodes for enzyme calpain 1. This variant was also associated with cardiomyopathies (*p*=0.005) and heart failure (*p*=0.005) in FinnGen. CAPN1 had heart enhanced interactions with ACTC1 and ACTN2, which participate in the formation of the actin cytoskeleton and are previously associated with hereditary cardiomyopathies [19, 20]. The calpains are calcium-activated intracellular cysteine proteases. The proteolytic activity of calpains is induced in ischemia/reperfusion when Ca^2+^ homeostasis is disturbed and this is thought to contribute to formation of myocardial injury [21]. CAPN1 mediates cardiomyocyte apoptosis via induction of endoplasmic reticulum stress [22]. The calpains are also implicated in the development of cardiac hypertrophy and fibrosis by being activated by stress stimuli mediated by hormones and integrins [23].

Dysfunction of sarcomeres is a hallmark of hypertrophic cardiomyopathy, in which hypertrophy is typically accompanied by fibrosis [24]. Two variants were found in genes affecting myosin and sarcomere function, encoding for myosin co-chaperones UNC45A and UNC45B. The variant in *UNC45A* was associated with cardiomyopathy (*p*=0.036) in FinnGen and the variant in *UNC45B* was associated with heart failure (*p*=0.049). These genes are responsible for correct folding of the myosin head domain and are necessary for myosin accumulation during myocyte development [25]. Both genes interact with heat shock proteins HSP90AA1 and HSP90AB1 which are chaperone proteins needed for proper myofibril assembly. Studies using *c.elegans* and drosophila models indicate that UNC45 may have an important role in sarcomere assembly and contractility of the heart [26, 27].

There are limitations to this study. We used the FinnGen dataset to investigate the role of these variants beyond the Fingesture study, validation in this independent dataset demonstrated significant associations with cardiomyopathies and heart failure, affirming the broader relevance of these variants in myocardial fibrosis beyond sudden cardiac death cases. Further replication in an independent dataset where myocardial fibrosis is confirmed would be necessary to verify the role of the identified variants and candidate genes in myocardial fibrosis.

To explore how each variant predisposes individuals to myocardial fibrosis and understanding the exact biological function of each variant and gene would require *in vitro* and *in vivo* experiments. The formation of myocardial fibrosis is a process effected by multiple factors besides the genetic predisposition. While we cannot infer the variant effect sizes based on this study, rare variants have the potential for moderate to large effects, therefore further studies are needed to elucidate the variants role in myocardial fibrosis [28]. The majority of the variants detected in this study can be viewed as variants of unknown significance (VUS) with clinical classifications such as the ACMG criteria, this is a known caveat of using large scale sequencing for autopsy victims noted also in the recent guidelines [29, 30]. These novel variants found in this study are therefore not to be used on their own in diagnostics or risk assessment in clinical practice and these results should be viewed as a search for new pathways in myocardial disease in SCD. The discovery of novel variants in PMF provides an opportunity to understand the processes driving myocardial fibrotic accumulations. It must be recognized that clinical classification such as ACMG criteria emphasize prior scientific reports regarding the variant pathogenicity estimation and these are bound to be nonexisting for possible novel genes and novel variants of a disease. Therefore, other methods of novel variant classification need to be used as we did in this study.

In summary, we identified novel variants associated with primary myocardial fibrosis in SCD victims. These variants, located in genes paritcipating in the function of extracellular matrix and cardiomyocyte activities, expand our understanding of the genetic underpinnings of myocardial fibrosis. Our results highlight the importance of ECM pathways, as well as of cardiomyocyte function and survival in the pro-fibrotic process.

## Supplementary information

ESM 1 (DOCX 22 kb)

ESM 2 (DOCX 14 kb)

ESM 3 (DOCX 17 kb)

ESM 4 (DOCX 31 kb)

## Data Availability

The data, analytical methods, and study materials will be made available to other researchers for the purposes of reproducing the results or replicating the procedure. Inquiries can be directed to the corresponding author. The Fingesture metadata is described in the national Finnish Fairdata service Etsin (https://etsin.fairdata.fi) and the reported variants are stored in the European Variation Archive (PRJEB47695, https://www.ebi.ac.uk/eva/).

## Abbreviations

PMF: Primary Myocardial Fibrosis
SCD: Sudden Cardiac Death
