# Supplementary material for: Novel Genetic Variants Associated with Primary Myocardial Fibrosis in Sudden Cardiac Death Victims
Source: J Cardiovasc Transl Res. 2024 Jun 7;17(6):1229–39. doi: 10.1007/s12265-024-10527-5 (PMC11634914; doi:10.1007/s12265-024-10527-5)
Supplement: Supplementary file 1 — (DOCX 22 kb) [file 12265_2024_10527_MOESM1_ESM.docx]

Supplementary Table 1. FinnGen endpoints of cardiac diseases. Association results for the variants identified in exome sequencing were extracted from the results of genome-wide association analyses from these endpoints.

| Endpoint phenocode in FinnGen | Endpoint description |
| --- | --- |
| I9_HEARTFAIL_AND_CHD | Heart failure and coronary heart disease |
| FG_CARDMYO | Cardiomyopathy (excluding other) |
| I9_CARDMYO | Cardiomyopathy |
| I9_HYPERTROCARDMYOP | Hypertrophic cardiomyopathy |
| I9_CARDMPRI | Cardiomyopathies, Primary/intrinsic |
| I9_HEARTFAIL_AND_OVERWEIGHT | Heart failure and bmi 25plus |
| I9_HEARTFAIL | Heart failure,strict |
| I9_HEARTFAIL_AND_HYPERTCARDIOM | Heart failure and hypertrophic cardiomyopathy |
| I9_CARDMYOHYP | Cardiomyopathy, Hypertrophic obstructive |
| I9_AF_REIMB | Atrial fibrillation and flutter with reimbursement |
| I9_HEARTFAIL_NS | Heart failure, not strict |
| I9_HEARTFAIL_ALLCAUSE | All-cause Heart Failure |
| I9_ISCHHEART | Ischemic heart diseases |
| I9_ANGINA | Angina pectoris |
| I9_IHD | Ischaemic heart disease, wide definition |
| I9_OTHARR | Other arrhytmias |
| I9_NONISCHCARDMYOP | Non-ischemic cardiomyopathy |
| I9_HEARTFAIL_AND_ANTIHYPERT | Heart failure and antihypertensive medication |
| I9_AVBLOCK | AV-block |
| I9_NONISCHCARDMYOP_STRICT | Nonischemic cardiomyopathy |
| I9_OTHHEART | Other heart diseases |
| I9_VHD | Valvular heart disease excluding rheumatic fever |
| I9_VALVES | Valvular operations |
| I9_CABG | Coronary artery bypass grafting |
| I9_MI_COMPLICATIONS | Complications following myocardial infarction |
| I9_SECONDRIGHT | Secondary right heart disease |
| I9_MYOCARD | Myocarditis |
| I9_PERICAOTH | Other diseases of pericardium |
| I9_NONRHEVALV | Non-rheumatic valve diseases |
| I9_CARDMYOALC | Alcoholic cardiomyopathy |
| I9_CARDMSEC | Cardiomyopathies, Secondary/extrinsic |
| I9_THAORTANEUR | Thoracic aortic aneurysm |
| I9_LBBB | Left bundle-branch block |
| I9_STR_EXH | Ischaemic Stroke, excluding all haemorrhages |
| Q17_ASD | Atrial septal defect |
| I9_CHD | Major coronary heart disease event |
| I9_TIA | Transient ischemic attack |
