# Supplementary material for: Novel Genetic Variants Associated with Primary Myocardial Fibrosis in Sudden Cardiac Death Victims
Source: J Cardiovasc Transl Res. 2024 Jun 7;17(6):1229–39. doi: 10.1007/s12265-024-10527-5 (PMC11634914; doi:10.1007/s12265-024-10527-5)
Supplement: Supplementary file 2 — (DOCX 14 kb) [file 12265_2024_10527_MOESM2_ESM.docx]

**Supplementary Table 2. Pathogenic variants in known candidate genes identified in the PMF cases.** Three individuals had a pathogenic variant in cardiomyopathy related candidate genes. These variants were each present in a single individual.

| Variant | Ref (NCBI) | Gene | Function | dbSNP | ClinVar | Phenotype |
| --- | --- | --- | --- | --- | --- | --- |
| c.G1083C>A;p.Trp361Ter | NM_022437 | *ABCG8* | nonsense | rs137852987 | Pathogenic/Likely pathogenic | Sitosterolemia 1 |
| c.3296dupG;p.Tyr1100fs*48 | NM_000256 | *MYBPC3* | frameshift insertion |  | Pathogenic | Hypertrophic cardiomyopathy |
| c.877C>T; p.Arg293Ter | NM_001122606.1 | *LAMP2* | nonsense |  | Pathogenic | Hypertrophic cardiomyopathy, Danon disease |
