# Supplementary material for: Novel Genetic Variants Associated with Primary Myocardial Fibrosis in Sudden Cardiac Death Victims
Source: J Cardiovasc Transl Res. 2024 Jun 7;17(6):1229–39. doi: 10.1007/s12265-024-10527-5 (PMC11634914; doi:10.1007/s12265-024-10527-5)
Supplement: Supplementary file 3 — (DOCX 17 kb) [file 12265_2024_10527_MOESM3_ESM.docx]

Supplementary Table 3 Results of the matrisome analysis

| Gene | Original AA | Variant AA | Original dipole | Variant dipole | Staining reliability | Heart-enhanced interactors |
| --- | --- | --- | --- | --- | --- | --- |
| CAPN1 | unique | aliphatic | - | - | Enhanced | ACTC1, ACTN2, AKT1, ATG5, CAPNS1, CAST, COL1A1, DES, ECHS1, FANCC, FANCG, FHL2, GAS2, HIF1A, MAPT, MYBPC3, NDUFB7, NFE2L1, PALB2, PRMT5, PTGDS, PTPN1, RAD21, SPTAN1, STAT3, TINAGL1, VCAM1, VIM, CTSC, GPT, SH3BGR, SLIT3 |
| CCL22 | aliphatic | acidic | - | +'+'+' | NA | CCR4 |
| CRTAC1 | basic | neutral | +++ | +3 | Enhanced | MAPRE2 |
| FRAS1 | aliphatic | aliphatic | - | + | Approved | none |
| HK2 | basic | neutral | +++ | +3 | Enhanced | VDAC1 |
| HPSE | basic | NA | +++ | NA | NA | none |
| LIMS1 | aliphatic | neutral | - | + | Approved | ACTB, ILK, KPRP, MAPT, NCK2, PARVA, SMURF1, STUB1, TCEA2 |
| MTUS2 | basic | aromatic | +++ | ++ | Enhanced | ABHD15, ANKRD11, AQP1, BARD1, C1orf35, CBX8, CDC73, CNNM3, CYSLTR2, DHRS1, DOCK2, ENPP7, FAM107A, FAM161A, FAM161B, FBXW5, GADD45GIP1, GAS2L2, GEM, IL16, KANK2, KAT5, KIF2C, KRT75, KRT76, LMO4, MAPRE1, MRPL11, NDOR1, NXF1, PPP1R18, PRKAA2, PRPF18, RANBP3L, RTP5, SDCBP, SLC25A42, SPATC1L, TACO1, TAF13, TCEA2, TCHP, THAP7, TNNI1, TRIM29, ZMAT2, ZNF414, ZNF439, ZNF440, ZNF572, ZNF580, ZNF792, ZNF512B, CEP95, CCDC151 |
| MYBPHL | basic | aromatic | +++ | ++ | NA | none |
| NMRK2 | unique | aliphatic | - | - | Approved | ST7 |
| NRIP1 | basic | neutral | +++ | + | Approved | ACTN2, AHR, BRCA1, CARM1, CIB1, CTBP1, CTBP2, DNMT1, DNMT3A, DNMT3B, E2F1, ESR1, ESRRA, FHL1, HDAC3, HDAC5, JUN, KPNB1, NR3C1, PRMT1, RXRA, THRA, XPO1, YWHAQ |
| OMA1 | acidic | aromatic | +'+'+' | + | Approved | none |
| PLB1 | aliphatic | aliphatic | - | + | NA | TSEN15 |
| SYT9 | aliphatic | aliphatic | - | - | NA | SYNCRIP, TUBB |
| TGM6 | neutral | aromatic | +3 | + | NA | none |
| TLR3 | aliphatic | aliphatic | - | - | Enhanced | BIRC2, BIRC3, CASP8, FADD, HLA-DRA, KCND2, KCND3, MAP2K6, MAP3K7, MYD88, PIK3R1, RIPK1, RNF31, SHARPIN, SYK, TAB2, TRAF6, TRIM69, UNC93B1, WDFY1 |
| TNS2 | unique | acidic | - | +'+'+' | Approved | AQP1, ARID5A, BLZF1, CNNM3, CPSF7, DZIP3, ENKD1, FASLG, KPRP, KRT76, KRTAP11-1, PHF1, PIN1, PLSCR3, PRR22, SERTAD2, SOCS7, SORBS3, TEKT3, TRIM8, USP54, ZNF575, ZNF134 |
| UNC45A | unique | neutral | - | +3 | Approved | HSP90AA1, HSP90AB1, TEKT1, FAM83H |
| UNC45B | basic | aromatic | +++ | ++ | NA | AHSA1, HSP90AA1, HSP90AB1 |
| VASN | basic | neutral | +++ | +3 | NA | CYSRT1, KRTAP11-1, KRTAP2-3, KRTAP2-4, KRTAP3-2, RGS19, TGFB1, VWC2 |
| WNT8B | unique | aliphatic | - | - | NA | none |
