# Supplementary material for: Novel Genetic Variants Associated with Primary Myocardial Fibrosis in Sudden Cardiac Death Victims
Source: J Cardiovasc Transl Res. 2024 Jun 7;17(6):1229–39. doi: 10.1007/s12265-024-10527-5 (PMC11634914; doi:10.1007/s12265-024-10527-5)
Supplement: Supplementary file 4 — (DOCX 31 kb) [file 12265_2024_10527_MOESM4_ESM.docx]

Supplementary Table 4. **Association results of variants identified in exome sequencing with cardiac disease endpoints in FinnGen database.** Association analyses were performed using SAIGE mixed models with age, sex and 10 PCs used as covariates. We examined the associations between the variants identified in exome analysis and cardiac disease endpoints in FinnGen. The genetic association results were derived from the FinnGen study database of GWAS summary statistics. A detailed description of all endpoints can be found from the FinnGen phenotype database (https://r7.risteys.finngen.fi/).

| **CAPN1; rs534135243** |  |  |  |  |  |  |  |
| --- | --- | --- | --- | --- | --- | --- | --- |
| **Endpoint** | **pval** | **beta** | **maf** | **maf_case** | **maf_control** | **n_case** | **n_control** |
| Heart failure and coronary heart disease | 0.00518239 | 0.561465 | 0.00118077 | 0.00190733 | 0.00115582 | 9463 | 275526 |
| Cardiomyopathy (excluding other) | 0.00542625 | 0.791825 | 0.00116883 | 0.00239449 | 0.00115063 | 3341 | 225036 |
| Cardiomyopathy | 0.00854024 | 0.662212 | 0.00118267 | 0.00217108 | 0.00116188 | 4606 | 218984 |
| Hypertrophic cardiomyopathy | 0.010283 | 1.20604 | 0.00117911 | 0.00371287 | 0.00117247 | 808 | 308346 |
| Cardiomyopathies, Primary/intrinsic | 0.0106928 | 0.74557 | 0.0011787 | 0.00231624 | 0.00116188 | 3238 | 218984 |
| Heart failure and bmi 25plus | 0.011245 | 0.52045 | 0.00120687 | 0.00165726 | 0.00116365 | 12397 | 129190 |
| Heart failure,strict | 0.0175918 | 0.392738 | 0.0011728 | 0.00150337 | 0.00114892 | 19676 | 272371 |
| Heart failure and hypertrophic cardiomyopathy | 0.021752 | 1.52123 | 0.00117609 | 0.00460123 | 0.00117247 | 326 | 308346 |
| Cardiomyopathy, Hypertrophic obstructive | 0.0265956 | 2.51 | 0.00116642 | 0.0034642 | 0.00116188 | 433 | 218984 |
| Atrial fibrillation and flutter with reimbursement | 0.0281618 | 0.445646 | 0.001123 | 0.00147673 | 0.00108314 | 17629 | 156457 |
| Heart failure, not strict | 0.0310299 | 0.267538 | 0.00117911 | 0.00140261 | 0.00114892 | 36783 | 272371 |
| All-cause Heart Failure | 0.0363036 | 0.345626 | 0.00117247 | 0.00145118 | 0.00115381 | 19350 | 288996 |
|  |  |  |  |  |  |  |  |
| **CCL22; rs777890031** |  |  |  |  |  |  |  |
| **Endpoint** | **pval** | **beta** | **maf** | **maf_case** | **maf_control** | **n_case** | **n_control** |
| Ischemic heart diseases | 0.00593035 | -0.388717 | 0.00115315 | 0.000935122 | 0.00119424 | 49030 | 260124 |
| Angina pectoris | 0.0127438 | -0.416189 | 0.00116798 | 0.000915361 | 0.00119424 | 27046 | 260124 |
| Ischaemic heart disease, wide definition | 0.0133217 | -0.333364 | 0.00115315 | 0.000966463 | 0.00118806 | 48706 | 260448 |
| Other arrhytmias | 0.0212403 | -0.40555 | 0.00116344 | 0.000831577 | 0.00119504 | 20851 | 218984 |
| Non-ischemic cardiomyopathy | 0.0246139 | -0.668388 | 0.00118806 | 0.000596555 | 0.00120451 | 7047 | 253401 |
| Heart failure and antihypertensive medication | 0.0478476 | -0.466248 | 0.00118785 | 0.000873995 | 0.00122666 | 18650 | 150798 |
| Heart failure,strict | 0.0486441 | -0.381714 | 0.00115606 | 0.000849946 | 0.00117817 | 19676 | 272371 |
|  |  |  |  |  |  |  |  |
| **CRTAC1; rs140424345** |  |  |  |  |  |  |  |
| **Endpoint** | **pval** | **beta** | **maf** | **maf_case** | **maf_control** | **n_case** | **n_control** |
| AV-block | 0.00253998 | -0.595484 | 0.005011 | 0.00296391 | 0.00504809 | 3968 | 218984 |
| Nonischemic cardiomyopathy | 0.0026641 | -1.24957 | 0.00497696 | 0.00134261 | 0.00499536 | 1310 | 258718 |
| Other heart diseases | 0.00465843 | -0.127731 | 0.00487861 | 0.00446703 | 0.00504809 | 90170 | 218984 |
| Valvular heart disease excluding rheumatic fever | 0.00494174 | -0.143872 | 0.0049055 | 0.00440365 | 0.00504809 | 62218 | 218984 |
| Valvular operations | 0.00556814 | -0.144118 | 0.00491236 | 0.00441145 | 0.00504809 | 59338 | 218984 |
| Other arrhytmias | 0.0142194 | -0.189965 | 0.00496798 | 0.00412667 | 0.00504809 | 20851 | 218984 |
| Coronary artery bypass grafting | 0.0151988 | -0.315348 | 0.00492069 | 0.00320497 | 0.00497803 | 8693 | 260124 |
| Atrial fibrillation and flutter with reimbursement | 0.0292409 | -0.225802 | 0.00489662 | 0.00406616 | 0.0049902 | 17629 | 156457 |
| Cardiomyopathies, Primary/intrinsic | 0.0318229 | -0.397787 | 0.00502082 | 0.00317674 | 0.00504809 | 3238 | 218984 |
|  |  |  |  |  |  |  |  |
| **FRAS1; rs201963922** |  |  |  |  |  |  |  |
| **Endpoint** | **pval** | **beta** | **maf** | **maf_case** | **maf_control** | **n_case** | **n_control** |
| Complications following myocardial infarction | 0.00561655 | 2.48532 | 0.000799034 | 0.00572368 | 0.000795759 | 173 | 260124 |
| Secondary right heart disease | 0.031012 | 2.26403 | 0.000777317 | 0.00194301 | 0.000774334 | 772 | 301704 |
| **HPSE; rs141973931** |  |  |  |  |  |  |  |
| **Endpoint** | **pval** | **beta** | **maf** | **maf_case** | **maf_control** | **n_case** | **n_control** |
| Secondary right heart disease | 0.0127186 | 0.740363 | 0.00742155 | 0.0129534 | 0.0074074 | 772 | 301704 |
| Myocarditis | 0.0177052 | -0.588834 | 0.00726748 | 0.00304169 | 0.00729962 | 1190 | 156457 |
|  |  |  |  |  |  |  |  |
| **LIMS1; rs145559017** |  |  |  |  |  |  |  |
| **Endpoint** | **pval** | **beta** | **maf** | **maf_case** | **maf_control** | **n_case** | **n_control** |
| Other diseases of pericardium | 0.0150477 | 1.23004 | 0.00275063 | 0.00567031 | 0.00274015 | 786 | 218984 |
| Non-rheumatic valve diseases | 0.0205282 | 0.282128 | 0.0027733 | 0.00323277 | 0.00274015 | 15799 | 218984 |
|  |  |  |  |  |  |  |  |
| **MTUS2; rs201600406** |  |  |  |  |  |  |  |
| **Endpoint** | **pval** | **beta** | **maf** | **maf_case** | **maf_control** | **n_case** | **n_control** |
| Alcoholic cardiomyopathy | 0.00501534 | 1.45915 | 0.00549156 | 0.0252525 | 0.00548513 | 99 | 304548 |
| Myocarditis | 0.0143357 | 0.580336 | 0.00549663 | 0.00888779 | 0.00547084 | 1190 | 156457 |
| Complications following myocardial infarction | 0.0172429 | 1.75078 | 0.00544774 | 0.0144509 | 0.00544176 | 173 | 260124 |
| Cardiomyopathies, Secondary/extrinsic | 0.0206605 | 2.00126 | 0.00550604 | 0.0162602 | 0.0055 | 123 | 218984 |
| Nonischemic cardiomyopathy | 0.0437794 | 0.535775 | 0.00544761 | 0.00840144 | 0.00543265 | 1310 | 258718 |
| Thoracic aortic aneurysm | 0.0447744 | 0.256177 | 0.00551801 | 0.00692526 | 0.00548934 | 5881 | 288638 |
|  |  |  |  |  |  |  |  |
| **NMRK2;rs146474422** |  |  |  |  |  |  |  |
| **Endpoint** | **pval** | **beta** | **maf** | **maf_case** | **maf_control** | **n_case** | **n_control** |
| Left bundle-branch block | 0.022867 | -0.730888 | 0.00415283 | 0.00115962 | 0.0041716 | 1373 | 218984 |
|  |  |  |  |  |  |  |  |
| **NRIP1; rs202001270** |  |  |  |  |  |  |  |
| **Endpoint** | **pval** | **beta** | **maf** | **maf_case** | **maf_control** | **n_case** | **n_control** |
| Other arrhytmias | 0.0370886 | -0.337652 | 0.00111746 | 0.000791611 | 0.00114849 | 20851 | 218984 |
|  |  |  |  |  |  |  |  |
| **SYT9; rs117876446** |  |  |  |  |  |  |  |
| **Endpoint** | **pval** | **beta** | **maf** | **maf_case** | **maf_control** | **n_case** | **n_control** |
| AV-block | 0.0320273 | 0.444957 | 0.00349842 | 0.00457879 | 0.00347885 | 3968 | 218984 |
|  |  |  |  |  |  |  |  |
| **TGM6; rs146485197** |  |  |  |  |  |  |  |
| **Endpoint** | **pval** | **beta** | **maf** | **maf_case** | **maf_control** | **n_case** | **n_control** |
| Heart failure and bmi 25plus | 0.020246 | -0.333527 | 0.00260308 | 0.00188803 | 0.0026717 | 12397 | 129190 |
|  |  |  |  |  |  |  |  |
| **TNS2; rs200670407** |  |  |  |  |  |  |  |
| **Endpoint** | **pval** | **beta** | **maf** | **maf_case** | **maf_control** | **n_case** | **n_control** |
| Ischaemic Stroke, excluding all haemorrhages | 0.00808984 | 0.308402 | 0.00224442 | 0.00286063 | 0.00220772 | 16857 | 283057 |
| Heart failure and bmi 25plus | 0.0141769 | 0.389549 | 0.00220726 | 0.00271302 | 0.00215873 | 12397 | 129190 |
| Atrial septal defect | 0.0190722 | 1.19864 | 0.00223408 | 0.00504036 | 0.00222635 | 843 | 305910 |
| Other diseases of pericardium | 0.0414467 | 1.13171 | 0.00219974 | 0.00445293 | 0.00219165 | 786 | 218984 |
| Heart failure and antihypertensive medication | 0.0464505 | 0.292797 | 0.00222148 | 0.00258918 | 0.00217601 | 18650 | 150798 |
|  |  |  |  |  |  |  |  |
| **UNC45A; rs146513919** |  |  |  |  |  |  |  |
| **Endpoint** | **pval** | **beta** | **maf** | **maf_case** | **maf_control** | **n_case** | **n_control** |
| Cardiomyopathy | 0.0360131 | -0.483724 | 0.00208999 | 0.00119409 | 0.00210883 | 4606 | 218984 |
| Cardiomyopathy (excluding other) | 0.0417763 | -0.548311 | 0.0020969 | 0.00104759 | 0.00211248 | 3341 | 225036 |
| Cardiomyopathies, Primary/intrinsic | 0.0487944 | -0.537724 | 0.00209385 | 0.00108091 | 0.00210883 | 3238 | 218984 |
|  |  |  |  |  |  |  |  |
| **UNC45B; rs141654082** |  |  |  |  |  |  |  |
| **Endpoint** | **pval** | **beta** | **maf** | **maf_case** | **maf_control** | **n_case** | **n_control** |
| Major coronary heart disease event | 0.0308056 | 0.28604 | 0.00123857 | 0.00145782 | 0.00121181 | 33628 | 275526 |
| Heart failure and bmi 25plus | 0.0491349 | -0.388153 | 0.00131108 | 0.000925429 | 0.00134808 | 12397 | 129190 |
|  |  |  |  |  |  |  |  |
| **VASN; rs148092711** |  |  |  |  |  |  |  |
| **Endpoint** | **pval** | **beta** | **maf** | **maf_case** | **maf_control** | **n_case** | **n_control** |
| Complications following myocardial infarction | 0.0213565 | 1.3112 | 0.00375829 | 0.0117874 | 0.00375295 | 173 | 260124 |
| Transient ischemic attack | 0.0250952 | 0.237407 | 0.00373787 | 0.00448208 | 0.00370161 | 13790 | 283057 |
